# Efficacy of cognitive enhancers for Alzheimer’s disease: protocol for a systematic review and network meta-analysis

**DOI:** 10.1186/2046-4053-1-31

**Published:** 2012-06-28

**Authors:** Andrea C Tricco, Sondra vanderVaart, Charlene Soobiah, Erin Lillie, Laure Perrier, Maggie H Chen, Brenda Hemmelgarn, Sumit R Majumdar, Sharon E Straus

**Affiliations:** 1 Li Ka Shing Knowledge Institute, St. Michael’s Hospital, 209 Victoria Street, East Building, Toronto, ON, M5B 1T8, Canada; 2 Division of Nephrology, Department of Medicine, University of Calgary, Calgary, AB, Canada; 3 Division of General Internal Medicine, Department of Medicine, University of Alberta, Calgary, AB, Canada; 4 Department of Geriatric Medicine, University of Toronto, Toronto, ON, Canada

## Abstract

**Background:**

Approximately 35 million people world-wide have Alzheimer’s disease and this is projected to nearly double by 2030. Cognitive enhancers, including cholinesterase inhibitors (for example, donepezil, galantamine and rivastigmine) and memantine (N-methyl-D-aspartic acid (NMDA) receptor antagonist) have been approved for the treatment of Alzheimer’s disease in many countries. Our objective is to evaluate the comparative effectiveness, safety, and cost of cognitive enhancers for Alzheimer’s disease through a systematic review.

**Methods/design:**

Studies examining the efficacy, safety, and cost of cognitive enhancers compared to placebo, supportive care, and other cognitive enhancers for Alzheimer’s patients will be included. The primary outcome is cognition and secondary outcomes include function, behavior, quality of life, safety, and cost. Experimental studies (randomized controlled trials, quasi-randomized controlled trials, controlled clinical trials), quasi-experimental studies (controlled before-after, interrupted time series), and observational studies (cohort, case–control studies) will be eligible for inclusion. Inclusion will not be limited by publication status, time period or language of dissemination.

We will search electronic databases (for example, MEDLINE, Cochrane Central Register of Controlled Trials, EMBASE, CINAHL, Ageline) from inception onwards. The electronic database search will be supplemented by searching for grey literature (for example, conference proceedings, searches in Google and relevant organization websites). Two reviewers will independently screen the studies for inclusion using the eligibility criteria established *a priori* and independently extract data. Risk of bias will be assessed using the Cochrane Risk of Bias tool for experimental and quasi-experimental studies and the Newcastle Ottawa Scale for observational studies. If deemed appropriate, meta-analysis and network (that is, indirect comparisons) meta-analysis will be conducted.

**Discussion:**

Our systematic review will inform the decision of healthcare providers, policy-makers, Alzheimer’s patients and family members about the use of cognitive enhancers, by improving their understanding of the costs, benefits and harms that are associated with these agents.

**PROSPERO registry number:**

CRD42012001948

## Background

In 2010, approximately 35 million people world-wide had Alzheimer’s dementia (AD). It has been projected that by 2030, this figure will nearly double to 65.7 million, and reach 115.4 million by 2050 [1]. In the United States, the economic burden of Alzheimer’s was $172 billion in 2010 and is estimated to soar to $1 trillion by 2050 [2]. AD is the most common cause of dementia [3]. It has an insidious onset with progressive deterioration in cognition, functional ability, behavior, and mood [4]. Patients living with AD have a lower quality of life and AD ultimately leads to death with a median survival of seven years from diagnosis [5]. Currently, there is no cure for AD.

The management of AD focuses on slowing progression, symptom control, maintaining functional status, improving quality of life, minimizing adverse events, and decreasing caregiver stress. Non-pharmacologic therapy includes social support, cognitive rehabilitation [6], assistance with activities of daily living, multidisciplinary programs [7], and providing support to caregivers [8]. The results of systematic reviews examining these management strategies have found marginal improvements in patient outcomes [6-8].

Recent cognitive enhancers for pharmacologic treatment for AD include the cholinesterase inhibitor drug class (donepezil, galantamine and rivastigmine), as well as memantine, a N-methyl-D-aspartic acid (NMDA) receptor antagonist [9]. The acetylcholinesterase inhibitors donepezil (Aricept, Eisai/Pfizer) and rivastigmine (Exelon, Novartis) have similar modes of action, increasing the concentration of acetylcholine at the neurotransmitter sites[10]. Galantamine (Reminyl, Shire) is another acetylcholinesterase inhibitor that increases acetylcholine at neurotransmitter sites, yet also acts by modulating activity at nicotinic receptors [10]. The NMDA receptor antagonist, memantine (Ebixa, Lundbeck), works on the glutamatergic system and modulates the neurotransmitter glutamate [10].

Although cognitive enhancers offer hope for patients with AD, high dropout rates have been observed in randomized trials of these agents and numerous adverse drug reactions (ADRs), including nausea, vomiting, diarrhea, syncope and bradycardia, have been seen with their use in practice [9]. None of these ADRs were identified as being a major issue in the seminal randomized clinical trials (RCTs) of efficacy [9]. Previously conducted systematic reviews of these agents have been limited by a lack of comparisons across all available medications; using restrictive inclusion criteria; and not selecting studies for inclusion, abstracting data or evaluating study quality in duplicate [11,12]. Furthermore, these systematic reviews did not examine adverse events from sources other than RCTs (for example, cohort studies), which would allow the identification of important ADRs. Of critical importance, one study found that hospitalization for bradycardia was associated with recent initiation of a cholinesterase inhibitor and that more than half of the patients who survived to discharge subsequently resumed therapy, highlighting that clinicians underappreciated the toxicity of this medication [13].

We are conducting this systematic review to determine the comparative effectiveness, safety, and costs associated with cognitive enhancers versus placebo, each other, or best supportive care for AD and severe AD. Furthermore, we wish to examine when each of the cognitive enhancers should be stopped due to lack of efficacy.

## Methods/design

This is a protocol for a systematic review, based on the PRISMA Statement [14], which was registered with the PROSPERO database (CRD42012001948).

### Eligibility criteria

We will include studies of elderly AD patients using cognitive enhancers approved for use in Canada (donepezil, rivastigmine, galantamine, memantine) compared with other cognitive enhancers, memantine or placebo and/or supportive care. Eligible studies include those with patients with mild, moderate or severe AD. Mild AD is defined as a score of 21 to 26 on the Mini-mental State Examination (MMSE) [9], moderate AD as an MMSE score of 10 to 20 and severe AD as an MMSE score <10. The studies must report validated measures to diagnose AD, including the Diagnostic and Statistical Manual of Mental Disorders (DSM) criteria and the National Minimum Data Set (NMDS) criteria. If the study includes patients with mixed dementia, it will be included if the predominant form of dementia is AD. Subgroups of interest that we will explore through subgroup analysis include severity of AD, previous response to treatment for AD, presence of behavioral disturbance, comorbid conditions (for example, stroke), and medication usage (for example, statins; baseline, dosage, and pattern of cognitive enhancers usage).

To be included in the analysis, the studies must report at least one of the following outcomes: cognition, function, behavior, quality of life, costs or harms. To further refine these outcomes, we engaged key stakeholders, including patients and their caregivers, healthcare providers, and policy-makers. This is a form of ‘integrated knowledge translation’ and we use a modified Delphi process [15] to establish consensus across key stakeholder groups. To facilitate the refinement of outcomes, we will follow the three steps for considering the relative importance of outcomes, as outlined by GRADE (Grading of Recommendations Assessment Development and Evaluation) [16]: preliminary classification of outcomes as critical, important but not critical or low importance before reviewing the evidence; reassessment of the relative importance of the outcomes after reviewing the evidence; and judgment of the balance between the desirable and undesirable effects of an intervention. The outcomes of interest (which will likely be rated differently by key stakeholder groups) include:

Cognition: measured by any valid scale including the MMSE, Alzheimer’s Disease Assessment Scale, Goal Attainment Scale, Severe Impairment Battery

Function: measured using any valid scale including Alzheimer’s Disease Cooperative Studies Activities of Daily Living Inventory, Alzheimer’s Disease Functional Assessment and Change Scale, Bristol Activities of Daily Living Scale, Caregiver-rated Modified Crichton Scale, Disability Assessment for Dementia, the Interview for Deterioration in Daily Living Activities in Dementia, Nurses Observation Scale for Geriatric Patients Activities of Daily Living subscale, the Progressive Deterioration Scale.

Behavior: measured by any valid scale including the Neuropsychiatric Inventory

Global Status: measured by any valid scale including Clinician Interview-Based Impression of Change Incorporating Caregiver Information scale, Clinical Global Impression of Change

Clinical Outcomes: Mortality, Health-Related Quality of Life, Institutionalization; harms (number of adverse events (for example nausea, vomiting, diarrhea, dizziness, weight loss, hospitalizations, bradycardia), number of withdrawals, number of withdrawals due to adverse events, severity and timing of adverse events); benefits to caregivers (for example caregiver stress).

Costs and cost effectiveness

The primary outcomes are cognition and function as measured by validated scales described above. Secondary outcomes include behavior, global status, clinical outcomes and costs.

We will include experimental studies (including RCTs, quasi-randomized trials, controlled clinical trials) and quasi-experimental studies (including interrupted time series and controlled before and after studies). To look for rare and unexpected adverse events and explore efficacy and clinical monitoring over time, we will also include observational studies (for example, cohort, case control studies). Inclusion will not be limited by publication status, time period or language of dissemination. Articles not written in English will be translated to determine their eligibility.

### Information sources and literature search

Literature search strategies will be developed using medical subject headings (MeSH) and text words related to cognitive enhancers for AD. The databases searched will include MEDLINE (OVID interface, 1946 onwards), EMBASE (OVID interface, 1947 onwards), Cochrane Central Register of Controlled Trials (CENTRAL; current issue), CINAHL (EBSCO interface, 1981 onwards), and Ageline (EBSCO interface, 1978 onwards).

The electronic database search will be supplemented by searching for grey literature (that is, difficult to locate or unpublished material). Specifically, we will search public health and trial registry websites (for example, Public Health Agency of Canada, Health Canada, FDA, *meta*Register of Controlled Trials), websites of organizations that produce guidelines (for example, Canadian Agency for Drugs and Technologies in Health, Center for Disease Control and Prevention, World Health Organization, Agency for Healthcare Research and Quality, National Institute for Health and Clinical Excellence), conference abstracts (International Pharmaceutical conference), and conduct general Internet searches in Google using key phrases and terms. Relevant journals (*Age and Aging* and the *Journal of the American Geriatrics Society*) will be hand searched from 1990 to the present. We will contact manufacturers to obtain their Scientific Information Packets for the medications. Reference lists of previous reviews on a similar topic will be scanned to identify further material [11,12]. Literature saturation will be ensured by searching the authors’ personal files, contacting manufacturers of cognitive enhancers, reviewing bibliographies from key retrieved articles, forward citation searching using Scopus and Web of Science, and contacting experts in the field, such as clinicians, researchers, and the Drug Safety and Effectiveness Network for Observational Studies.

An experienced librarian (LP) will conduct the literature searches. The search strategy will be peer reviewed by another librarian using Peer Review of Electronic Search Strategies (PRESS) [17]. The draft literature search can be found in Additional file 1. The results from the literature search will be uploaded to our online SysRev Tool [18]. This software will be used for screening the citations resulting from the search, as well as all full-text articles identified through the search.

### Study selection process

To ensure reliability, a training exercise will be conducted prior to commencing screening. Using the inclusion and exclusion criteria, a random sample of 50 citations from the literature search will be screened by all reviewers. Inter-rater agreement for study inclusion will be calculated using percent agreement and the kappa statistic [19]. If poor to moderate agreement is observed (that is, percent agreement less than 70% or a kappa statistic less than 0.6), the inclusion and exclusion criteria will be clarified to facilitate consistent application of the selection criteria by the research team (for example, we may need to clarify that AD does not include Lewy Body Dementia for the non-clinical reviewers involved with this project). Reviewers will only abstract data when the kappa statistic is greater than 0.6. Each citation will be screened by two independent reviewers using the pre-specified inclusion and exclusion criteria. Potentially relevant full-text articles will be obtained and screened by two independent reviewers. Conflicts will be resolved by discussion or the involvement of a third reviewer.

### Data items and data collection process

The data abstracted will include study characteristics (for example, study design, year of trial conduct, sample size, setting, country of study conduct, intervention and comparator details), participant characteristics (for example, type and number of patients, age mean and standard deviation, AD diagnosis criteria, AD severity, baseline cognition, co-morbidities), and outcome results (for example, cognition, function, behavior, quality of life, costs, and harms). The data will be extracted using the online SysRev Tool. The online form will be piloted and will be further refined, as necessary, if poor agreement is observed. Specifically, we will review data abstraction elements contributing to the low agreement and clarify the wording in the data collection forms to ensure that the data abstractors are interpreting them in a similar fashion. To ensure data accuracy, two reviewers will independently abstract all of the data and discrepancies will be resolved by discussion or the involvement of a third reviewer.

We suspect that in some instances studies will report outcome results over many different time periods. We will abstract data from each time period to examine the effects of the interventions on the relevant outcomes over time. Healthcare providers and policy-makers have noted that the timepoints of greatest interest are at 6, 12 and 24 months in our discussions with them. Furthermore, many studies follow patients to three months and we will also include this timepoint in our analysis.

We also suspect that multiple study publications may report data from the same study group (that is, companion reports). When this occurs, the report with the critical outcomes of interest will be included and used to abstract data. The other report(s) will provide supplementary data only. We will contact the study authors for further information when the data are not clearly reported; this is particularly important for outcomes data because outcomes that are positively influenced by treatment are more likely to be reported [20].

### Methodological quality/risk of bias appraisal

We will appraise the methodological quality/risk of bias using standardized quality assessment tools for design-specific internal validity. For RCTs, we will use the Cochrane Risk of Bias Tool [21]. For controlled clinical trials, interrupted time series, and controlled before-after studies, we will use the Cochrane Effective Practice and Organisation of Care Risk of Bias Tool [22]. For cohort studies and case control studies, we will use the Newcastle-Ottawa Scale [23]. Subsequently, we will use GRADE to create a summary of findings tables and to assess the level of evidence across studies. Publication bias will be assessed using funnel plots [24].

Development of instruments for assessing risk of bias in studies of harms is still in the early stages [25]. Santaguida and colleagues have developed a quality rating tool for evaluating studies reporting harms based on a review of the literature (called McHarm) [26]. It has been tested for face and construct validity and we will use it in conjunction with other standardized quality assessment tools.

### Synthesis of included studies

The systematic review results will first be described narratively and, where possible, pooled estimates of effects will be derived using a random-effects model [27]. Meta-analysis will be performed separately for cognitive enhancers versus placebo or best supportive care, against each other, and versus memantine for severe AD. We will not statistically combine the results of different study designs in the meta-analysis. If the outcome is continuous, then mean difference and its 95% confidence intervals will be used. If the outcome is binary, then the odds ratio will be used when observational studies are assessed and the risk ratio will be used when trials are analyzed.

When meta-analysis is conducted, we will assess for clinical, statistical, and methodological heterogeneity. We will look at the forest and funnel plots to assess for obvious heterogeneity based on visual inspection. We will also quantitatively assess heterogeneity; if extensive heterogeneity is observed (for example, a statistically significant chi-squared test (*P* < 0.1) for heterogeneity or an I^2^ statistic greater than 60%) [28], we will conduct meta-regression analysis. The meta-regression analysis will explore the influence of factors such as age, co-morbidities (for example, chronic conditions), and baseline effect sizes on the meta-analysis results. Meta-regression will be done when ten or more studies are available [29]. Both meta-analysis and meta-regression will be analyzed using SAS 9.2 [30].

We anticipate that some of the studies will not report all relevant data and to include them in the analysis, we will impute missing data using established methods [31]. We will conduct sensitivity analysis to examine the effect of this method using an approach proposed by Carpenter *et al*. [32], which entails imputing missing data under a missing at random assumption, and then reweights the imputed data to allow for nonrandom selection.

If the data allow, network (that is, indirect comparison) meta-analysis will be conducted by using WinBUGS (MRC Biostatistics Unit, Cambridge, England) to derive the combined outcome between two treatments as well as rank the efficacy among all available treatment arms [33]. WinBUGS is a Bayesian software program used to build complex statistical models using the Markov chain Monte Carlo method. To facilitate the practicality of treatment comparisons, median rankings will be used as point estimations of treatment efficacy. A random effects model with indirect/mixed treatment comparison based on the WinBugs code derived by Ades *et al*. [33] will be used for network meta-analysis. To distinguish between significant and non-significant treatment efficacies, 95% credible intervals (CIs) will be established using the 2.5 and 97.5 percentiles obtained via Monte Carlo simulation of 10,000 iterations. We will interpret the 95% CI as being equivalent to confidence intervals derived from frequentist methods [33]. We will base decisions about combining studies on thorough investigations of clinical and methodological diversity as well as variation in effect size [Fu *et al*.]. We will do this by re-examining information in the reports on some trials, calculating direct and indirect estimates separately before proceeding to a network meta-analysis [34].

To assess whether results are robust to trial design/study quality, sensitivity analyses will be performed by excluding trials with high rates of participant exclusions where losses are considered to have the potential to impact on the results. Sensitivity analysis will also be conducted on imputing missing data (as described above), instrument used for the primary outcomes example (we will look at each of the validated instruments used for assessing cognition separately if they are used in three or more trials), average adherence between groups (we will look at studies that reported the average adherence and examine those that reported the lower adherence rate and the higher adherence rate), and the impact of including observational studies in the analyses (we will combine trials and observational studies for this analysis only). Since network meta-analysis is based on the Bayesian approach and is sensitive to the priors used in the model, we will conduct sensitivity analysis using different priors for variance parameters [35].

## Discussion

Our systematic review results have the potential to influence a large proportion of the population. Of the more than 0.5 million Canadians with dementia, 30% of this group will have moderate dementia and two-thirds of these will have AD indicating that more than 100,000 patients are eligible for cognitive enhancers. However, in 2004 more than 900,000 prescriptions for cognitive enhancers were filled and $129 million was spent on these agents in Canada. The number of prescriptions and money spent on cognitive enhancers is only going to increase over time as the proportion of people with AD continues to increase.

To ensure widespread dissemination of our results, we will employ a multi-faceted knowledge translation strategy. Firstly, we will publish our results in an open access journal so that our results are widely available. Secondly, we will present our findings at relevant meetings such as those of the Cochrane Collaboration, Canadian Geriatrics Society, American Geriatrics Society, and so on. Thirdly, we will disseminate our results through newsletters of interested organizations, such as the Drug Safety and Effectiveness Network in Canada, and the Alzheimer’s Society among others. Fourthly, to facilitate changes in knowledge, attitudes and behaviors of family physicians we will link with continuing education providers to create an education module. In addition, we will use our results to inform the development of a patient decision aid and will create summary sheets of key results for clinicians and healthy policy-makers, increasing the likelihood that our results will be used. Lastly, we will disseminate findings to social/mass media for mainstream uptake.

## Competing interests

The authors declare they have no competing interests.

## Authors’ contributions

ACT conceived the study, designed the study, helped obtain funding for the study, and helped write the draft protocol. SV helped write the draft protocol. LP developed the search strategies and edited the draft protocol. CS registered the protocol and edited the draft protocol. EL edited the draft protocol. MHC provided input to the design and draft of the protocol. BH and SM provided feedback on the protocol during its development. SES conceived the study, designed the study, obtained the funding, and helped write the draft protocol. All authors read and approved the final protocol.

## Supplementary Material

Additional file 1 Literature search. Click here for file
