# Supplementary material for: Efficacy of cognitive enhancers for Alzheimer’s disease: protocol for a systematic review and network meta-analysis
Source: Syst Rev. 2012 Jun 28;1:31. doi: 10.1186/2046-4053-1-31 (PMC3407718; doi:10.1186/2046-4053-1-31)
Supplement: Additional file 1 — Literature search. [file 2046-4053-1-31-S1.doc]

**Appendix 1: Literature search**

Database: Ovid MEDLINE(R) <1948 to November Week 2 2011>, Ovid MEDLINE(R) In-Process & Other Non-Indexed Citations <November 22, 2011>

Search Strategy:

--------------------------------------------------------------------------------

1. alzheimer$.mp.
2. "benign senescent forgetfulness".mp.
3. binswanger$.mp.
4. (chronic adj2 cerebrovascular).mp.
5. (cognit$ adj2 (impair$ or declin$ or deficit$ or degenerat$ or deteriorat$ or los$ or disorder$ or complain$ or disturb$)).mp.
6. (cerebr$ adj2 (impair$ or declin$ or deficit$ or degenerat$ or deteriorat$ or los$ or disorder$ or complain$ or disturb$)).mp.
7. (memory adj2 (impair$ or declin$ or deficit$ or degenerat$ or deteriorat$ or los$ or disorder$ or complain$ or disturb$)).mp.
8. (mental adj2 (impair$ or declin$ or deficit$ or degenerat$ or deteriorat$ or los$ or disorder$ or complain$ or disturb$)).mp.
9. (ne?rocognit$ adj2 (impair$ or declin$ or deficit$ or degenerat$ or deteriorat$ or los$ or disorder$ or complain$ or disturb$)).mp.
10. (ne?ro-cognit$ adj2 (impair$ or declin$ or deficit$ or degenerat$ or deteriorat$ or los$ or disorder$ or complain$ or disturb$)).mp.
11. ((cognit$ or memory or cerebral or brain) adj2 (improv$ or enhanc$ or perform$ or process$ or function$ or rehabilitation or aid$ or stimulat$)).mp.
12. cognition.ti.
13. (confusion$ or confused).tw.
14. dement$.mp.
15. deliri$.mp.
16. "ischemic white matter".mp.
17. ("normal pressure hydrocephalus" and shunt$).mp.
18. "organic brain disease$".mp.
19. "organic brain syndrome".mp.
20. presenil$.tw.
21. pre-senil$.tw.
22. senil$.tw.
23. Alzheimer Disease/
24. Cognition Disorders/
25. Cognition/de [Drug Effects]
26. Confusion/
27. Delirium, Dementia, Amnestic, Cognitive Disorders/
28. Delirium/
29. Dementia/
30. Memory Disorders/
31. (aMCI or MCI).mp.
32. ("AA CD" or AACD).tw.
33. ("AA MI" or AAMI).tw.
34. ARCD.tw.
35. ("CI ND" or CIND).tw.
36. LCD.tw.
37. (MCD or MNCD).tw.
38. "M-MCI".tw.
39. "N-MCI".tw.
40. or/1-39
41. abixa.tw.
42. aricept.tw.
43. (acetylcholinesterase adj inhibitor$).mp.
44. axura.tw.
45. akatinol.tw.
46. anti-cholinesterase?.tw.
47. anticholinesterase?.tw.
48. (cognitive adj enhanc$).mp.
49. (cholinesterase adj inhibitor$).mp.
50. ChEI.tw.
51. donepezil.mp.
52. ebixa.tw.
53. eranz.tw.
54. exelon.tw.
55. galant?amin$.tw.
56. lycoremine.tw.
57. memantin$.tw.
58. memox.tw.
59. namenda.tw.
60. nimvastid.tw.
61. nivalin$.tw.
62. "N-Methyl-D-aspartic acid receptor antagonist$".tw.
63. prometax.tw.
64. razadyne.tw.
65. reminyl.tw.
66. rivastigmine.mp.
67. exp Cholinesterase Inhibitors/
68. Galantamine/
69. Memantine/
70. 357-70-0.rn. [CAS Registry Numbers]
71. 19982-08-2.rn.
72. 120011-70-3.rn.
73. 120014-06-4.rn.
74. 123441-03-2.rn.
75. or/41-74
76. 40 and 75
77. Animals/ not (Animals/ and Humans/)
78. 76 not 77
